# Supplementary material for: Oxidation of DJ-1 Cysteines in Retinal Pigment Epithelium Function
Source: Int J Mol Sci. 2022 Sep 1;23(17):9938. doi: 10.3390/ijms23179938 (PMC9456479; doi:10.3390/ijms23179938)

Figure S1. DJ-1 sequence, and predesigned shRNA constructs used in this manuscript. **A)** NCBl Homo sapiens Parkinson disease 7 (PARK7) mRNA sequence with highlighted target sequences of shRNA constructs used. **B**) Table of the predesigned PARK7 shRNA used and their target sequence. **C**) Representative western blot of lysates of ARPE-19 clones transfected PARK7 shRNA and probed with DJ-1and GAPDH.

A

ATGGCTTCCAAAAGAGCTCTGGTCATCCTGGCTAAAGGAGCAGAGGAAATGGAGACGGTC

(2)

ATCCCTGTAGATGTCATGAGGCGAGCTGGGATTAAGGTCACCGTTGCAGGCCTGGCTGGA

(3)

AAAGACCCAGTACAGTGTAGCCGTGATGTGGTCATTTGTCCTGATGCCAGCCTTGAAGAT

GCAAAAAAAGAGGGACCATATGATGTGGTGGTTCTACCAGGAGGTAATCTGGGTGCACAG

AATTTATCTGAGTCTGCTGCTGTGAAGGAGATACTGAAGGAGCAGGAAAACCGGAAGGGC

CTGATAGCCGCCATCTGTGCAGGTCCTACTGCTCTGTTGGCTCATGAAATAGGTTTTGGAA

GTAAAGTTACAACACACCCTCTTGCTAAAGACAAAATGATGAATGGAGGTCATTACACCTAC

(4)

TCTGAGAATCGTGTGGAAAAAGACGGCCTGATTCTTACAAGCCGGGGGCCTGGGACCAG

(1)

CTTCGAGTTTGCGCTTGCAATTGTTGAAGCCCTGAATGGCAAGGAGGTGGCGGCTCAAGT

GAAGGCTCCACTTGTTCTTAAAGAC

B

Gene Symbol	Gene Description	TRC ID	Clone Name	Target Sequence
PARK7	Parkinson disease (PD, autosomal recessive, early onset) 7	TRCN0000004918	NM_007262.3-583s1c1	**GCAATTGTTGAAGCCCTGAAT**
PARK7	PD autosomal recessive, early onset) 7	TRCN0000004919	NM_007262.3-169s1c1	**GCTGGGATTAAGGTCACCGTT**
PARK7	PD autosomal recessive, early onset) 7	TRCN0000004920	NM_007262.3-221s1c1	**GTAGCCGTGATGTGGTCATTT**
PARK7	PD autosomal recessive, early onset) 7	TRCN0000004921	NM_007262.3-506s1c1	**ACTCTGAGAATCGTGTGGAAA**

C

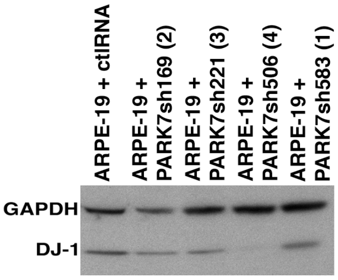

Figure S2. Transduction and characterization of ARPE-19 monolayers with the DJ-1 adenoviruses. **A)** Quantitative RT-PCR analysis of CMV promoter present in adenoviruses constructs. **B**) Representative images of ARPE-19 monolayers transduced with the control (Ad), human DJ-1 (hDJ-1), human DJ-1 with the cysteine at residues 46, 53 and 106 mutated to serine (C2S), human DJ-1 with the cysteine at residues 46 mutated to serine (C46S), human DJ-1 with the cysteine at residues 53 mutated to serine (C53S), and human DJ-1 with the cysteine at residues 106 mutated to serine (C106S) adenoviruses and probed with an antibody to adenovirus type 5 (red) and TO-PRO3 (nuclei, blue). Bar = 20 µm.

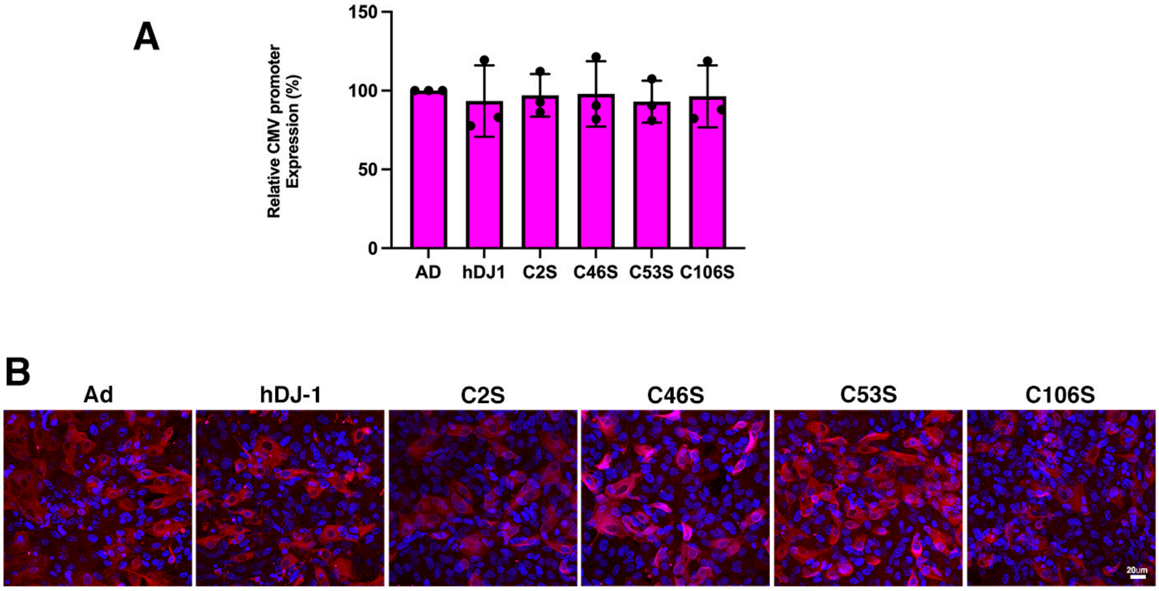

Supplement: Supplementary file 1 [file ijms-23-09938-s001.zip › ijms-1794805-supplementary.pdf]
